# Managerial Decision-making for Daily Case Allocation Scheduling and the Impact on Perioperative Quality Assurance

**Published:** 2016

**Authors:** Minmin Zhu, Zhengli Yang, Xiao Liang, Xiaojie Lu, Gurmukh Sahota, Renyu Liu, Lihua Yi

**Affiliations:** 1 Department of Anesthesiology, Wuxi Second Hospital, Nanjing Medical University; 2 Department of Nurse, Wuxi Second Hospital, Nanjing Medical University; 3 Department of Neurosurgery, Wuxi Second Hospital, Nanjing Medical University; 4 Department of Anesthesiology and Critical Care, Perelman School of Medicine at the University of Pennsylvania; 5 Center of Administration Office, Wuxi Second Hospital, Nanjing Medical University

**Keywords:** allocation scheduling, perioperative period, risk management, quality assurance, shift supervisor, chief resident

## Abstract

Allocation scheduling for daily surgical cases is a decision-making process tasked to anesthesiologists and nurse managers in the operating room (OR). This manuscript focuses on three major areas: the classification and principles of allocation scheduling on workdays in China, flexible strategies of operational decision-making given differences in planned versus actual OR allocations, and perioperative quality implications of anesthesia scheduling. Improved quality and optimal decision-making in daily surgical case scheduling is seen with shift supervisor-based scheduling of staff and cases when compared with staff and case scheduling managed by the departmental director or chief resident.

## 1. Introduction

Anesthesia has long been considered a pioneer in patient safety.(1) Every aspect of routine anesthetic tasks—such as urgent resuscitation, physiologic monitoring, and hemodynamic management—requires accurate judgment, comprehensive analysis, and rapid but appropriate responses to evolving and many times critical situations.(2) These necessary traits of anesthesiologists impact their success in avoiding danger and recovering from physiological insults that ultimately impact patient outcomes.

Several factors can increase the inherent risk in providing anesthesia to patients. Concomitant disease may impair the reserved function of vital organs, making a thinner or even absent margin for any errors. Multiple medications, especially potentially lethal drugs, administered in a short interval, may interfere with organ system function. Finally, the surgical procedures themselves are physiologic stressors, even with anesthesia.(3–5)

These complicating conditions always challenge the anesthesia providers in terms of maintaining risk-reduction strategies, comprehensive analysis abilities, and performing emergency procedures. In part, anesthesia-related mortality is declining due to advances in monitoring technology, establishment and application of anesthesia regulations, and implementation of systematic measures to reduce human error.(6, 7) However, with the ultimate goal of improving patient safety, there is still room for improvement in the quality of anesthesia care and reduction of anesthesia-related perioperative risk.

Quality of medical care is an common theme in hospital management.(8) A major goal of quality improvement initiatives is to establish an integrated, standardized and systematic medical quality management system via a feedback process.(9) Quality improvement in anesthesia encompasses many aspects of the daily workflow that affect the process of anesthesia care and reduce the risk of anesthesia related injury. (10)

In the routine activities of medical facilities, scheduling of surgical cases is indispensable to maintain the normal function of operating rooms (ORs). Appropriate schedule management could reduce complication rates and increase efficiency of perioperative management, which plays an important role in the provision of overall services of the hospital.(11–13)

OR scheduling covers two different levels: monthly staff scheduling and daily allocation scheduling (daily staff assignment).(14) By definition, the former refers to long-term scheduling, whereas the latter refers to day-to-day function. Staff scheduling is the process of deciding which staff is available on a given day for a given shift. (15) There are 4 defined shifts: day shift, night shift before midnight, night shift after midnight, and the emergency shift. Monthly staff scheduling usually is created in advance—that is, the daily roster of available staff for each shift over the period of an entire month is determined at the end of the preceding month or even earlier.

A monthly scheduling table operates according to certain rules, ensuring each staff member is clear about their assigned role each day. In terms of the entire department, a scheduling table provides information about the working hours of each staff member on each day and ensures coverage of every shift with specified staff. The day shift implements a standard eight-hour workday. There are standardized working hours for the emergency and night shift both during the work days or on the weekends, but due to the unpredictable nature of resuscitation, emergent consultation, and the management of emergent surgical cases, accurate pre-planning of staff requirement is difficult.(16) For the night shifts and emergency shift, the dual-duty mode of staffing with two professional members (associate specialist anesthetists or senior anesthetists, junior anesthetists, or residents) for both emergency and night duties, has been adopted overwhelmingly in most hospitals.

Daily allocation scheduling describes the process of deciding who will take care of a specific patient on a specific day and nurses should ideally make such decisions.(18) These arrangements are based on surgical case volume and the number of staff available on the day shift. This task includes allocating surgical cases to specific ORs, accommodating changing priorities and monitoring of the case progression in real-time.(19)

This paper focuses on the perioperative quality implications of certain principles of OR scheduling such as flexible decision-making strategies with respect to continuously changing OR needs and anesthesia staffing requirements. We demonstrate the advantage of allocation scheduling by the shift supervisor, compared with the practice of scheduling by the department director or chief resident(s).

## 2. Types of Allocation Scheduling on Workdays

Allocation scheduling is generally split into planned scheduling and flexible arrangements. Planned scheduling is completed at noon on the day prior to the surgery. Flexible arrangements are adjusted based on the changes in the dynamic OR schedule on the day of surgery.

### 2.1. Planned scheduling

Planned scheduling is based on the intended surgical procedures listed and delivered, either in print or electronic form, from surgical departments to the OR management team.(20) (Note: Lists of elective surgery are usually delivered in the morning prior to the operation; notice of emergency surgery is usually provided about one hour prior to the operation.) The room number of each OR, the order of procedures, and equipment nurse(s) and circulating nurse(s) in each OR are arranged by OR personnel (usually the head nurse). The designated staff member, such as the shift supervisor, arranges the anesthesia staff who will take care of the patient(s) in each OR.

Elective surgery is usually organized according to certain rules, commonly applied in the sequencing of operations in the OR daily schedule.(21) Usually, the surgical cases are allocated based on the available OR time for specific surgical departments (or surgical group). Additionally, priority is given to certain procedures—most commonly, surgeries for children, surgeries with the shortest processing time, and surgeries with the longest processing time (these are performed either first or last). Outpatient surgeries generally are scheduled together, to be performed either first or last in the scheduling day(22). The same group of surgeons performs consecutive operations, with cases that have the highest likelihood of potential contamination, last. (23) Within the same operating theater, the procedures should be arranged in the order of surgical incision: type I incision operation (clean surgery), type II incision operation (relatively clean surgery), type III incision operation (clean-contaminated surgery), and type IV incision operation (contaminated surgery).

As a general guideline, the purpose of planned scheduling is to ensure that available resources are used efficiently and effectively to achieve the designated goals(19).

On the day of surgery, decision-making by ordered priorities would usually simplify organizational activities and reduce patient and surgeon waiting time. The accuracy of planned scheduling depends on reliable estimation of the duration of the surgery.(24) The planned sequence is often interrupted in actual practice by various unpredictable factors: i) the number of surgical cases and duration of operation procedures among different departments can vary greatly; ii) consecutive operations performed by multiple groups in the same OR are constrained by the need to share equipment and resources; iii) delay can occur between consecutive operations with a longer turnover time caused by the surgeons, anesthetists, nurses, patient transfer, and other factors.

### 2.2. Flexible arrangements

Generally, the original scheduling plan could be adjusted for the following scenarios:

1. *Changes in surgery procedures.* Cancelled or delayed surgery on the day of the procedure is usually due to inadequate patient preoperative preparation. For example, if concomitant disease is found before anesthesia, it will be necessary—because of concerns for patient safety—to improve the cardiopulmonary function reserve of patients to reduce the perioperative risk. The surgical plan may also change due to special situations—such as a prolonged (due to surgical complications) or shortened (for palliative treatment) operation or even cancellation of the operation, resulting in delay (if the operation time is much longer than expected) or underutilized OR time (when the actual time is shorter than expected or planned).
2. *Multiple simultaneous emergency cases.* In this situation, in addition to the emergency personnel on duty bearing the brunt of the unscheduled activities, alternative staff should be assigned.

## 3. Principles of Planned Scheduling and Adjustment Strategy for Flexible Arrangements on Workdays

In practice, most of the policy-making or strategy adjustments should follow these general principles: i) Perform all scheduled cases unless case cancellation is required because of a patient safety concerns; ii) reduce over-utilized OR time; or iii) reduce patient and staff waiting times(18).

In most hospitals, there are three surgery scheduling rules: i) a service should complete its elective cases within its allocated OR time; ii) a service should re-schedule its cases if the service cannot finish its scheduled case within its allocated OR time to avoid overbooking and over-utilized OR time; iii) a service could negotiate with a different service for a potentially overbooked case to allocate the case in that different service’s under-utilized OR time in order to avoid overall poor OR time utilization (both under and over utilization).(17)

The adjustment strategy for flexible arrangements requires modifying the original plan when the changes occur, including updating the order of surgery priority and providing staff flexibly to match the existing tasks, rather than vice versa(17). When changes occur—on the premise of a high percentage of procedures completed on time, high utilization of resources and equipment, and low overtime—there should be minimal disruption to the ORs and surgical staff. This practice assists in realizing the sequenced scheduling decisions and the stability of the task implementation.

Overall, the purpose of flexible arrangements is to rationalize the use of ORs and maximize their efficiency by: i) minimizing under-utilized OR time; ii) minimizing the hours of over-utilized OR time for anesthetists and nurses on the day of surgery (creating more predictable work hours and reducing the risk associated with case handoffs); iii) reducing wait-time for patients on a given day means reducing total tardiness of all patients scheduled in the OR, thus speeding up the turnover of patients—for emergency cases, this also means reducing patients’ waiting for availability of surgeons and other staff.(18)

## 4. Decision-making for Allocation Scheduling in the Anesthesiology Department on Workdays

In addition to matching the tasks and workload assigned to each OR with the working schedule of the facility’s anesthesiologists, the selection of eligible anesthesiologists generally follows these principles:

- *Rigorous subspecialty matching assignment.* There are multiple differing factors with regards to the practical conduct of anesthesia, complexity of intraoperative management, and variability of crisis situations in the clinical setting; therefore strict subspecialty assignment is one of the important safeguard measures to reduce risks. The purpose of the matching practice is to allocate specific tasks—such as anesthesia for cardiopulmonary bypass surgery, manipulation of double-lumen endobronchial tube and endobronchial blocker tube, complex cases in the critically ill individual and crisis management of anesthesia outside of ORs—to competent members whose anesthetic technique should be recognized as appropriate for the individual patient and for the proposed type of conduct of anesthesia.
- *Team dynamics.* The anesthesiologists requested by patients or doctors will be assigned to related procedures preferentially. This arrangement helps to reduce patients’ concerns, create a reassuring atmosphere for medical treatment, and reflect humane care. Surgeons, anesthesia practitioners, and nurses comprise a multidisciplinary team that is jointly responsible for the safety of surgical patients. Moreover, effective communication is the foundation of teamwork, and cooperation act as the key to ensuring safety and preventing crisis.(10) So, a good team means mutual support and cooperation, responsibly sharing and caring to achieve a common goal.
- *Continuity of care.* The anesthetists on the day shift or emergency shift in the surgery workday will be scheduled for severe and complex cases (such as non-cardiac surgery of patients with cardiac disease, aged or obese patients, and patients with severe organ dysfunction) on the following day, because preoperative visits need sufficient time to focus on and carefully evaluate the particular conditions and concomitant diseases that may increase the technical difficulty and potential risks during anesthesia.(25) If patients with special conditions require consultation, the anesthetist who has attended the consultation will be responsible for anesthetic management of these surgical cases. This arrangement is conducive to a more in-depth and global evaluation of patients before anesthesia, and in addition, it avoids handoffs.
- *Consideration of special factors.* If an anesthetist is pregnant or lactating, she should be free from anesthetic work in orthopedic surgery or interventional surgery, to avoid radiation. Proper consideration of working hours and work arrangements should be given to staff members who need to attend hospital meetings. For the novice anesthetist, tasks assigned should follow an easy-to-difficult sequence according to the technical anesthetic requirement and intraoperative management.
- *Research needs.* Appropriate anesthesia tasks are assigned to staff members with ongoing research projects, creating conditions for them to safely care for patients while also being able to fulfill the research program within the designated time limit.

## 5. Task-managing for Allocation Scheduling in the Anesthesiology Department on Workdays (Figure 1)

### 5.1. Quality improvement

#### 5.1.1. Concerns about workload and risk factors

The increasing size of hospitals leads to a rapid increase in the number of surgical cases.(26) In addition, the growing complexity of surgical procedures, non-surgical procedures, and interventional therapy have extended the breadth of clinical anesthesia applications. Moreover, as a result of the aging of the global population and changes in lifestyle, the increased proportion of high-risk surgery patients with advanced age, obesity, hypertension, diabetes, coronary heart disease, and other concomitant diseases; anesthetic care has become more complex. The OR staff can perceive that the desire to perform as many operations in a day as possible is so pervasive as to be the most dominant organizational factor underlying unsafe practices at work.(27) In most cases, raising the number of surgeries runs the risk of reducing the quality of care. Explicit or implicit, production pressure should not supersede patient safety and quality of medical care.

Scheduling should be fair and reasonable. Firstly, for flexible arrangements involving multiple ORs, decision-making to reduce over-utilized OR time often conflicts with decisions to increase clinical work per unit time in each OR.(18) Secondly, in most general hospitals in China, there is limited specialization and sub-specialization, thus anesthesiologists provide services for a variety of surgical departments for a variety of procedures. Anesthetic concerns vary based on American Society of Anesthesiologists (ASA) Physical Status Classification and surgical procedure. In order to ensure reasonable allocation of personnel, balanced tasks and equal practice opportunities should be assigned to each staff member, avoiding uneven distribution. This balancing of tasks and opportunities helps promote staff development and increase satisfaction.

#### 5.1.2. Evaluation of individual performance and limits

Anesthesia represents a critical care field because the anesthetized patient can be considered intrinsically at risk of severe physiologic disturbance. Anesthesiologists are expected to be skilled, knowledgeable, careful when monitoring the patient directly and via monitors, and prepared to correctly manage crisis conditions(1). As for safety, a high degree of vigilance and focus on details is extremely important. That high degree of vigilance requires that the anesthesiologist maintain situational awareness even when he/she has other tasks in progress.

Attention to detail means the anesthesiologist is able to concentrate on certain events even when he/she is acutely aware of the numerous surrounding stimuli. The anesthesiologist should observe anesthetic apparatus, monitors, patients, the surgical field, and the surrounding environment as a whole, constantly evaluating the dynamic interplay, and making differential diagnoses for a variety of events observed. Any abnormality should be validated by other independent methods (e.g., the heart rate based on the ECG can be verified by palpation or pulse oximetry), and simultaneous changes in different variables should be checked (for example, dramatic changes in heart rate could be accompanied by changes in blood pressure).

Distraction, fatigue, haste, sleep deprivation, and many other factors can cause errors. Awareness of human limitations should shape the work environment and patient care systems to make them both more functional and efficient. Organizational strategies—including supervision, preoperative evaluation, equipment improvement, additional monitoring, and mutual compensation—have been proposed to cope with these limits and help reduce stress.(1) Therefore, on the premise of completion of clinical tasks and orderly arrangement of work, task allocation should prevent the staff from working while fatigued and simultaneously ensure the continuity of care.

#### 5.1.3. Enhancing awareness of risk prevention and management

Anesthesia risk management is an important part of perioperative safety and quality assurance.(28) As opposed to staff from other clinical departments, anesthesiologists have phased participation in assigned tasks. The anesthetic implementation process (aka. manipulation, monitoring, and management of the patient’s vital signs along with intraoperative judgment) is basically completed by a single individual on many occasions. This requires the managers of an anesthesia department to adopt effective risk management strategies to circumvent anesthesia risks, improve perioperative healthcare quality, and guarantee the safety of patients.(29)

In the management of safety and quality assurance, multiple impact factors, such as the ability of supervision, flexibility, and variability of assigned tasks; configuration of limited resources (related to manpower, time, and equipment); particular status of the patients; complexity of anesthesia management; diverse skills required for anesthesia practitioners—must all be taken into account. Traditional anesthesia education has focused on the acquisition of expertise and the mastering of technical skills; however, non-technical skills such as task management, teamwork, situational awareness, and decision-making play a decisive role in risk reduction and management in anesthesia. Anesthesia risk management ideally should combine situation awareness and interpersonal communication skills along with professional knowledge and skills.(30)

### 5.2. Ongoing management

#### 5.2.1. Resource optimization

The rational allocation of planned scheduling and timely adjustment of flexible scheduling can achieve optimal utilization of available resources (equipment, personnel, and time). As the day progresses, the anesthesia manager monitors the progress of surgical cases in real-time and evaluates and coordinates changes in a timely fashion.

Flexible arrangements involve the reallocation and re-planning of ORs, personnel, expected working hours, workload, and so on. By reducing the underutilized time of ORs, both anticipated over-utilized time and mandatory emergency over-utilized time are reduced. Through timely release of anesthesia time, the flexibility of staff arrangements can be combined with variability of the plan to achieve successful management.

#### 5.2.2. Efficiency improvement

Rationalized allocation of resources, in the context of decision-making, help to achieve optimal efficiency and promote sustainable development. Orderly implementation of planned scheduling and timely coordination of flexible arrangements reduce the total duration of over-utilized OR time and, as a result, reduce expected and unexpected overtime and provide direct benefits. Economically rational completion of all scheduled cases each day results in minimized total over-utilized OR time and serves to maximize the ratio of output/input (clinical income/personnel costs), which means maximized OR efficiency(18).

#### 5.2.3. Running smoothly

The four distinct, interrelated, and simultaneous functions of successful management—planning, organizing, leading, and controlling—form a loop process that is essential to the smooth running of ORs. Planned scheduling establishes a global perspective of the situation to achieve the overall established objectives, reflecting the organization and leadership of management.(31) On the other hand, flexible arrangements pay attention to details and process, reflecting a refined and pragmatic style of management. Optimal decision-making effects can be achieved by optimizing processes that keep the department running smoothly.

## 6. Empiric Evidence of Allocation Scheduling in the Anesthesiology Department on Workdays (Table 1)

### 6.1. Chief resident-based decision-making

Decision-making management led by the chief resident (CR) is the mode commonly employed in many large hospitals.(32) The CR position is often seen as a bridge between attending physicians and senior residents, and this transitional stage generally lasts for one year. Individuals selected for chief are seen as resident leaders who have considerable authority over matters of concern to the house staff. The CR often balances multiple responsibilities, ranging from administrative duties such as scheduling junior residents, to patient care, research, and teaching medical students and fellow residents.(33) The CR can deal with planning and orderly and flexible adjustment; however, a low grade of seniority and inadequate experience may create a situation in which the CR lacks control and management capabilities and has difficulty performing a supervisory role in clinical activities.

### 6.2. Department director-based decision-making

This is a more traditional mode for a decision-making management system. The department director usually shoulders overall tasks in the department, including clinical work, discipline construction, personnel training, scientific research, education activity, and department efficiency, and other activities. The department director is able to take all factors into consideration to create overall plans for task arrangements.(34) However, other administrative duties often comprise a relatively large percentage of his/her time, leading to some difficulty in real-time tracking of every detail in the OR and this sometimes leads to a disconnect or omission in management.

### 6.3. Shift supervisor-based decision-making

This is a new type of decision-making management system. Individuals are selected as shift supervisors from anesthesiologists with senior positions (or senior professional titles) who had consultative experience in supervising. They are seen as leaders second only to the departmental director. From the management viewpoint, the shift supervisor is under the leadership of a department director. This subdivides the management unit in the department and makes the management framework more flat at three levels—i.e., hospital, department director, and shift supervisor—which is conducive to the implementation of regulations, target programs, and technology application at every level.

We have very positive experiences since the shift supervisor management system was implemented in Wuxi Second Hospital in May 2010. The positive experiences include not only efficient running of operating rooms—they also advantages in every level of management of anesthesia quality and perioperative safety note below.

In contrast to the chief resident, the shift supervisor consists of the high-performing anesthesia group as an executive committee to make decisions. First, the shift supervisor can strengthen quality control. He or she has the power to identify high-risk situations, apply intraoperative measures and technical pearls, and articulate a clear plan for the management of intraoperative emergencies. For the daily arrangements, according to the summary of preoperative visits provided in the morning meeting there are several additional aspects the shift supervisor can perform to improve patient care:, i) the more complex cases can get special attention to them during the critical portions of the procedure; ii) strengthen key components in the management process; iii) schedule required activities such as endotracheal intubation, extubation, and staff breaks; iv) solve problems in a timely manner and eliminate potential risks; v) ensure that the management of the post-anesthesia recovery unit effectively guarantees the smooth performance of subsequent procedures and recovery practices.

To ensure dynamic and ongoing departmental function, successful management of an OR system requires the leader to have knowledge of the organizational structure and operations of the institution and work environment. This includes the ability to set goals for and with staff under their domain and in other departments and the skills to work cooperatively and collaboratively with others.(35–37) Under the leadership of an experienced shift supervisor with skill in identifying and utilizing resources and allocating tasks to appropriate members of various departments, a hospital’s senior management team can focus on the target task management and smooth operation of the ORs on an ongoing basis.

The shift supervisor facilitates direct oversight (as a supervising consultant anesthetist present or only seconds away on the same site) to ensure that every aspect of anesthetic management is conducted competently. In the allocation of administrative responsibilities between the director and the shift supervisor, the director has overall responsibility for rules and regulations, administrative management, implementation of the mandatory tasks allotted from the hospital, and evaluation of the outcomes to meet goals and objectives.(38) The shift supervisor’s management responsibilities, in contrast, have emphasis on quality assurance of medical care and technical supervision. During the monthly shift, shift supervisors have overall responsibility for enabling appropriate measures to be taken to ensure the safety of patients and staff, communicating with patients and coordinating with other departments, organizing preoperative consultation of critical cases, and drafting anesthesia plans. The daily specific tasks include: routine anesthesia inspections of ORs, allocation scheduling for anesthesia arrangements, consultation within and outside of the hospital, and organization of temporary emergency teams’ responses to unexpected events.

In addition, the shift supervisor facilitates real-time feedback to achieve rapid decision-making in the face of continuing change. Although many discussions at the OR control desk involve decisions that affect multiple ORs, management by the shift supervisor ensures accurate real-time OR information feedback in routine inspections. Optimized decision-making with regards to flexible arrangements reduces deviation from scheduled start times, diminishes unoccupied OR time, and assists in assessing and streamlining discharge time of patients in the post-anesthesia care unit. In routine anesthesia inspections, the shift supervisor will make better dynamic coping strategies and timely adjustments according to integrated information for ever-changing situations, aiming at improving surgeons’ and patients’ satisfaction. The running of the department will be enhanced if the department is considered as a whole, global coordination is achieved, and the staffs at all levels are empowered to play an active role.

## 7. Conclusion

Shift supervisor-based decision-making for staff and case scheduling is beneficial in providing improved quality and optimal decision-making in daily surgical cases scheduling when compared with staff and case scheduling managed by the departmental director or chief resident.

## Figures and Tables

**Figure 1:**
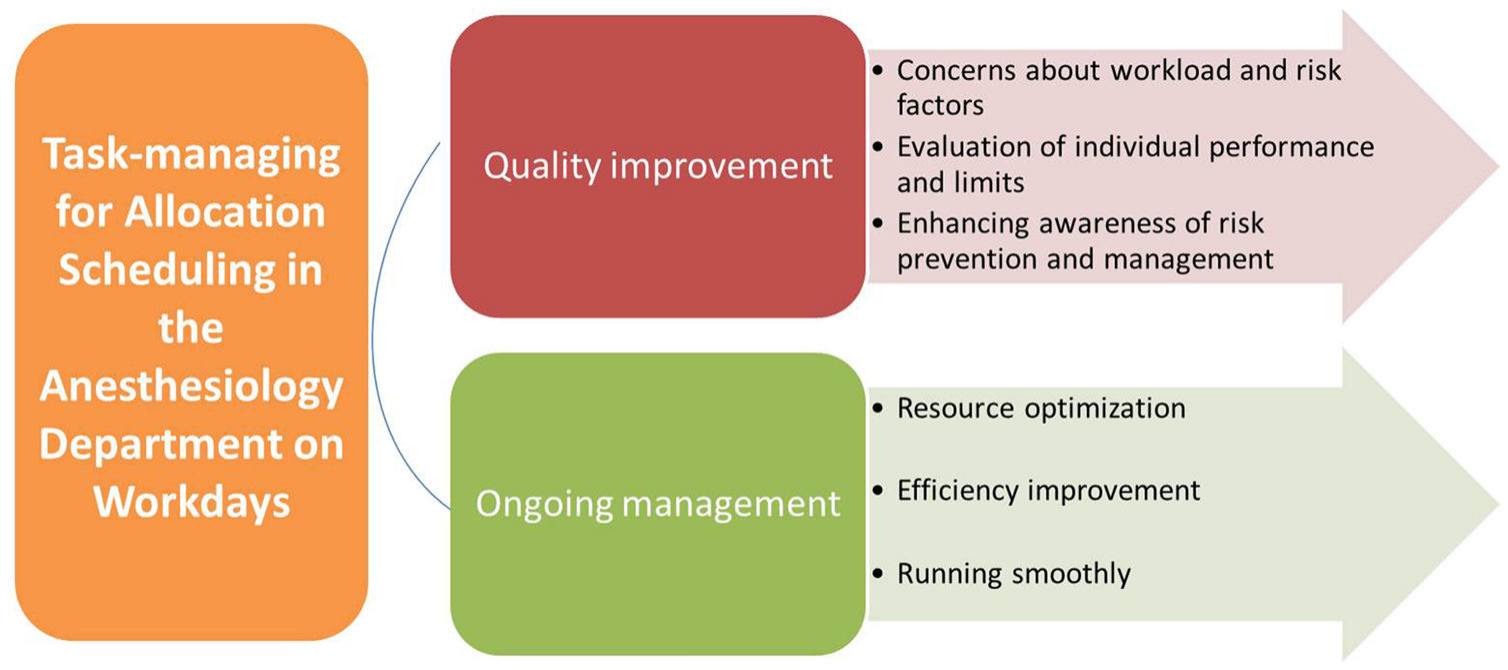
Task-managing for allocation scheduling in the anesthesiology department on workdays.

**Table 1:** Comparisons of different Role-based Decision-makings on OR Quality Assurance

Personnel	Shift cycle	Safety of OR Running			Efficiency of OR Running	
		Supervisory role in clinical activities	Overall tracking performance	Security patrol for quality control	Timely flexible arrangement	Coordination with other surgical teams
Chief resident	One year (more likely to be fatigued)	X	X	✓	✓	Weak
Department director	Every year (more vulnerable to pressure from management)	✓	X	X	X	Strong
Shift supervisor	One month (during the off-duty period, engage in front line work, does not necessitate leaving clinical work)	✓	✓	✓	✓	Strong
